# Amyloid-β Homeostasis Bridges Inflammation, Synaptic Plasticity Deficits and Cognitive Dysfunction in Multiple Sclerosis

**DOI:** 10.3389/fnmol.2017.00390

**Published:** 2017-11-21

**Authors:** Mario Stampanoni Bassi, Sara Garofalo, Girolama A. Marfia, Luana Gilio, Ilaria Simonelli, Annamaria Finardi, Roberto Furlan, Giulia M. Sancesario, Jonny Di Giandomenico, Marianna Storto, Francesco Mori, Diego Centonze, Ennio Iezzi

**Affiliations:** ^1^Unit of Neurology & Unit of Neurorehabilitation, IRCCS Istituto Neurologico Mediterraneo (INM) Neuromed, Pozzilli, Italy; ^2^Multiple Sclerosis Research Unit, Department of Systems Medicine, University of Rome Tor Vergata, Rome, Italy; ^3^Service of Medical Statistics & Information Technology, Fondazione Fatebenefratelli per la Ricerca e la Formazione Sanitaria e Sociale, Rome, Italy; ^4^Neuroimmunology Unit, Institute of Experimental Neurology (INSpe), Division of Neuroscience, San Raffaele Scientific Institute, Milan, Italy; ^5^Department of Clinical and Behavioural Neurology, IRCCS Santa Lucia Foundation, Rome, Italy; ^6^Clinical Pathology Unit, IRCCS Istituto Neurologico Mediterraneo (INM) Neuromed, Pozzilli, Italy

**Keywords:** amyloid-β, hippocampus, synaptic plasticity, inflammatory cytokines, IL-8, IL-10, IL-1ra

## Abstract

Cognitive deficits are frequently observed in multiple sclerosis (MS), mainly involving processing speed and episodic memory. Both demyelination and gray matter atrophy can contribute to cognitive deficits in MS. In recent years, neuroinflammation is emerging as a new factor influencing clinical course in MS. Inflammatory cytokines induce synaptic dysfunction in MS. Synaptic plasticity occurring within hippocampal structures is considered as one of the basic physiological mechanisms of learning and memory. In experimental models of MS, hippocampal plasticity is profoundly altered by proinflammatory cytokines. Although mechanisms of inflammation-induced hippocampal pathology in MS are not completely understood, alteration of Amyloid-β (Aβ) metabolism is emerging as a key factor linking together inflammation, synaptic plasticity and neurodegeneration in different neurological diseases. We explored the correlation between concentrations of Aβ_1–42_ and the levels of some proinflammatory and anti-inflammatory cytokines (interleukin-1β (IL-1β), IL1-ra, IL-8, IL-10, IL-12, tumor necrosis factor α (TNFα), interferon γ (IFNγ)) in the cerebrospinal fluid (CSF) of 103 remitting MS patients. CSF levels of Aβ_1–42_ were negatively correlated with the proinflammatory cytokine IL-8 and positively correlated with the anti-inflammatory molecules IL-10 and interleukin-1 receptor antagonist (IL-1ra). Other correlations, although noticeable, were either borderline or not significant. Our data show that an imbalance between proinflammatory and anti-inflammatory cytokines may lead to altered Aβ homeostasis, representing a key factor linking together inflammation, synaptic plasticity and cognitive dysfunction in MS. This could be relevant to identify novel therapeutic approaches to hinder the progression of cognitive dysfunction in MS.

## Introduction

Cognitive deficits are common in Multiple Sclerosis (MS), affecting almost half of the patients and negatively influencing social functioning and quality of life (Rao et al., 1991; Benedict et al., 2006; Chiaravalloti and DeLuca, 2008). MS is a chronic inflammatory immune-mediated disorder of the central nervous system (CNS) characterized by a variable course of clinical manifestations. Whereas the role of demyelinating white matter lesions and gray matter atrophy in motor and sensory deficits has been extensively investigated, the pathogenesis of cognitive dysfunction in MS is not completely elucidated. In recent years, neuroinflammation is emerging as a main factor possibly influencing cognitive dysfunction in MS (Gentile et al., 2015).

Different mediators released by immune cells, including inflammatory cytokines and neurotrophins, influence synaptic transmission. It has been proposed that an imbalance between proinflammatory (e.g., interleukin-1β, IL-1β, and tumor necrosis factor α, TNFα) and anti-inflammatory (e.g., IL-4 and IL-10) cytokines with a prevalence of the former, may contribute to brain damage in MS (Linker et al., 2005; Zeis et al., 2008; Ivanov and Lindén, 2009).

Different animal models of MS, including virus-induced (Theiler’s murine encephalitis virus induced demyelinating disease, TMEV-IDD) and autoimmune models (Experimental Autoimmune Encephalomyelitis, EAE) have been used to explore pathophysiological mechanisms of disease. EAE is induced by either the administration of protein or peptide in adjuvant or by the adoptive transfer of encephalitogenic T-cell blasts into naïve recipients. TMEV belongs to the cardiovirus group of the Picornaviridae and induces persistent immune demyelinating disease in mice (Miller, 1995). A common feature of both animal models is the release of proinflammatory cytokines and the recruitment of Th1 cell, monocytes and macrophages in the CNS, leading to myelin damage (Dal Canto and Lipton, 1975).

In EAE specific proinflammatory cytokines, including IL-1β and TNFα, alter both excitatory and inhibitory transmission resulting in synaptic hyperexcitability and excitotoxic neuronal damage (Centonze et al., 2009; Rossi et al., 2011; Mandolesi et al., 2013). Accordingly, the administration of either AMPA receptor inhibitors (Centonze et al., 2009) or IL-1β receptor antagonist (IL-1ra; Furlan et al., 2007; Mandolesi et al., 2013) is able to reduce both neurodegeneration and synaptic alterations in EAE mice, confirming the role of inflammation-induced excitotoxicity.

Also in MS patients, cerebrospinal fluid (CSF) levels of inflammatory cytokines are associated to analogous alterations of both inhibitory and excitatory transmission, resulting in synaptic hyperexcitability (Rossi et al., 2012a; Mori et al., 2016). Furthermore, CSF from MS patients in the active phase of disease reproduced in rodent brain slices both glutamatergic and GABAergic alterations and neuronal degeneration observed in EAE (Rossi et al., 2012a,b). Notably, these synaptic alterations did not occur when CSF from MS patients was coincubated with IL-1β inhibitors (Rossi et al., 2012a,b). In addition, different anti-inflammatory cytokines showed neuroprotective effects by normalizing glutamate (Garg et al., 2009), enhancing GABA signaling (S-Rózsa et al., 1997) and attenuating glutamate-mediated excitotoxicity (Zhou et al., 2009). Coherently, there is also evidence that anti-inflammatory cytokines may contribute to reduce synaptic hyperexcitability and neurodegeneration in MS patients (Rossi et al., 2011).

Overall, these data suggest that synaptic alterations associated to neuroinflammation may represent a critical factor inducing neuronal dysfunction in MS.

## Cognitive Deficits in MS and EAE

Different mechanisms have been proposed to explain cognitive impairment in MS patients. Clinical and magnetic resonance imaging (MRI) studies showed an association between white matter lesions and neuropsychological performance evaluated with different tests. In particular, lesion volume and site influence cognitive performance, highlighting the role of disconnection mechanisms (Vellinga et al., 2009; Kincses et al., 2011; Rossi F. et al., 2012). Gray matter damage is increasingly regarded as a main predictor of cognitive dysfunction in MS. Cortical lesions and in particular hippocampal CA1 region atrophy, has been associated with memory deficits in MS patients (Sicotte et al., 2008; Calabrese et al., 2009). In some cases, cognitive deficits appear already in the early phase of MS (Zivadinov et al., 2001; Olivares et al., 2005; Deloire et al., 2006) and are not associated to any substantial neuronal damage, namely isolated cognitive relapses (Coebergh et al., 2010; Pardini et al., 2014), suggesting that alternative mechanisms may be implicated. Moreover, the fact that cognitive impairment may occur at the early stage of the disease, before motor dysfunction appearance, suggests that cognitive decline associated with MS is mediated by a distinct mechanism, e.g., neuroinflammation.

Cognitive deficits have also been investigated in experimental models of MS. It has been demonstrated that spatial learning and memory deficits appear in EAE mice in different disease stages. In the late phase, for example, one study reported persisting memory acquisition and maintenance deficits after recovery of motor symptoms associated to reduced choline acetyltransferase activity in the hippocampus, cerebral cortex and basal forebrain. As memory deficits improved after anticholinesterase treatment, it was suggested that altered acetylcholine transmission could affect memory in the late phase of EAE (D’Intino et al., 2005). In addition, hippocampal degeneration and spatial learning deficits have been observed in EAE mice at a relatively late phase associated with decreased hippocampal volume and loss of GABAergic interneurons (Ziehn et al., 2010). These findings suggest that hippocampal structures seem to be particularly susceptible to inflammatory-dependent damage in EAE (Yirmiya and Goshen, 2011).

Learning and memory deficits have been also evidenced in the earlier phases of the disease, before the onset of motor and sensory symptoms, when EAE is induced in apolipoprotein E (APOE) knockout mice and human APOE ε4 (APOE4) knock-in mice (Tu et al., 2009). In particular, as early cognitive deficits and hippocampal cholinergic dysfunction were evident only in the presence of both EAE associated neuroinflammation and APOE-KO/APOE4 knock-in, a two-hit mechanism has been proposed. That is, increased susceptibility of cognitive deficit can be secondary to poor repair mechanisms associated with APOE-KO/APOE4 knock-in in the presence of a proinflammatory response (Tu et al., 2009). Interestingly, it has been recently suggested that APOE could play a critical role in neurodegenerative disorders regulating microglial function and promoting the switch to a neurodegenerative phenotype (Krasemann et al., 2017). APOE is the major apolipoprotein in the CNS, and is critically involved in neurite and synapse remodeling and synaptic plasticity (Kim et al., 2009). The APOE4 polymorphism has been associated with earlier age of onset in AD patients (Kim et al., 2009) and also associated with learning and memory deficits in MS, particularly in young patients (Shi et al., 2008). However, it should be noted that two studies found no association between APOE4 and cognitive deficits in MS (Portaccio et al., 2009; Carmona et al., 2011), therefore the role of this polymorphism in MS still requires further investigation.

## Synaptic Plasticity in MS and EAE

Synaptic plasticity occurring within hippocampal structures is considered as one of the basic mechanisms of learning and memory processes (Stuchlik, 2014). It can be hypothesized that, even in the absence of apparent anatomical damage, functional alterations in the hippocampus could disrupt synaptic plasticity leading to cognitive deficits.

The ability of neurons to undergo functional long-term modifications at existing synapses is referred to as synaptic plasticity. Long-term potentiation (LTP), one of the most studied form of synaptic plasticity, consisting in a persistent enhancement of synaptic strength, is also characterized by structural rearrangements and neurotrophin-induced protein synthesis (Bliss and Collingridge, 1993; Cunningham et al., 1996; Murray and Lynch, 1998; Malenka, 2003). Another form of synaptic plasticity, known as long-term depression (LTD), describes long-lasting weakening of synaptic strength. Different experimental protocols have been designed to explore LTP and LTD-like plasticity in EAE animal model and in MS patients.

Preclinical studies in EAE showed that inflammatory cytokines alter synaptic plasticity. Impairment of hippocampal LTP has been reported during the initial acute phase in EAE and has been associated with a selective reduction of NMDA receptors, microglial activation and IL-1β increase (Di Filippo et al., 2013). Moreover, persistent microglial activation together with impaired hippocampal LTP was also shown during remission in EAE (Di Filippo et al., 2016). Conversely, other studies evidenced that inflammation may also subvert synaptic plasticity. A study, exploring both LTP and LTD-like hippocampal plasticity in EAE, showed that LTP induction was favored over LTD. In particular, this alteration was mediated by the proinflammatory cytokine IL-1β, interfering with GABAergic transmission (Nisticò et al., 2013). Moreover, *in vivo* blockade of IL-1β in EAE reduced the alterations of hippocampal synaptic plasticity (Mori et al., 2014). Altogether, these data show that inflammation alters hippocampal synaptic plasticity *in vitro* in EAE mouse model.

Synaptic plasticity can be also explored non-invasively in MS patients by using specific transcranial magnetic stimulation (TMS) protocols (Mariorenzi et al., 1991; Fitzgerald et al., 2004; Ziemann et al., 2008). In particular, two different theta burst stimulation (TBS) protocols have been widely used to elicit LTP-like and LTD-like effects, respectively intermittent TBS (iTBS) and continuous TBS (cTBS; Di Lazzaro et al., 2005; Huang et al., 2005).

TMS studies evidenced that in relapsing remitting (RR)-MS patients, CNS inflammation alters plasticity (Stampanoni Bassi et al., 2017) and that, during relapses, iTBS induced LTP-like plasticity is impaired (Mori et al., 2011, 2012). In remitting patients, response to the iTBS protocol was comparable to healthy controls. Conversely, in response to cTBS an abnormal LTP-like effect was observed, which showed a positive correlation with CSF IL1-β levels (Mori et al., 2014). It has been proposed that, during relapses, the lack of LTD-like effects after cTBS may rely on reduced GABAergic transmission (Caramia et al., 2004; Rossi et al., 2012b) or increased glutamatergic signaling (Rossi et al., 2012a).

These results suggest that synaptic plasticity is profoundly altered by neuroinflammation, providing a plausible substrate for cognitive deficits. In particular, some evidence suggests that acute inflammation can be associated with both cognitive impairment and altered synaptic plasticity (Mori et al., 2011, 2012). In RR-MS patients, acute inflammation, as evidenced by the presence of gadolinium enhancing (Gd+) lesions at MRI scan, was associated with both impaired LTP-like plasticity and cognitive impairment, as shown by reduced PASAT score (Mori et al., 2012). According with the inflammatory origin of these alterations, both PASAT score and synaptic plasticity improved after 6-month treatment with interferon-β (IFN)-beta 1a in Gd+ patients, whereas they did not in patients without evidence of acute inflammation at MRI (Mori et al., 2012).

## Inflammation and Amyloid-β Metabolism

LTP expression could be regulated by different dynamics acting both at receptor level and on downstream mechanisms triggered upon receptor activation (Bliss and Collingridge, 1993; Kessels and Malinow, 2009; Minichiello, 2009). Amyloid-β (Aβ) modulate synaptic functioning through different mechanisms including the modulation of other signaling systems (cytokines, neurotrasmitters/messengers), in particular the modulation of nitrergic system and involvement of IL-1 receptors could play a crucial role (Morgese et al., 2015). An emerging key factor linking together inflammation, synaptic plasticity and neurodegeneration in different neurological diseases is the alteration of Aβ metabolism. Aβ peptides derive from the proteolytic cleavage of amyloid precursor protein (APP), a transmembrane protein. In the amyloidogenic pathway, APP undergoes a first cleavage by beta-site APP-cleaving enzyme 1, followed by further cleavage by γ-secretase to release 40 or 42 amino-acid long Aβ fragments. However, APP may go through different non-amyloidogenic pathways preventing Aβ formation (Andreasson et al., 2007). The Aβ peptides are highly hydrophobic and tend to aggregate to form dimers, oligomers or amyloid fibrils and have been identified as a major insoluble component of amyloid plaques (Masters and Selkoe, 2012). The aggregation of soluble oligomers to insoluble fibrils in amyloid plaques of Alzheimer’s disease (AD) reduces the Aβ CSF concentrations (Blennow and Hampel, 2003).

Several experimental data support the view that Aβ can impair hippocampal LTP (Yamin, 2009). Injections of Aβ oligomers in rats inhibit LTP (Walsh et al., 2002) and promote LTD (Li et al., 2009) in the hippocampus. Moreover, it has been shown that Aβ_1–42_ can alter both early and late LTP phases (Chen et al., 2002; Zhao et al., 2004). Altered Aβ metabolism has been consistently associated to the pathophysiology of AD and in particular with hippocampal LTP impairment (Klyubin et al., 2005; Shankar et al., 2008). Accordingly, Aβ dimers isolated from AD patients can impair hippocampal LTP and memory in mice and induce dendritic spine retraction in neurons (Shankar et al., 2008). Although through different pathophysiological mechanisms, brain inflammation is a common feature of both MS and AD (Lassmann, 2011). Under normal physiological conditions, there is a balance between Aβ production and clearance (Iwata et al., 2001; Saito et al., 2005) and inflammation can alter such equilibrium (Griffin et al., 2006; Hickman et al., 2008; Schmidt et al., 2008). Therefore, as in AD, Aβ may represent a possible player influencing both synaptic dysfunction and neurodegeneration occurring in MS as well (Gentile et al., 2015). Indeed, Aβ can be found in MS multifocal lesions (Ferguson et al., 1997; Trapp et al., 1998). Furthermore, reports of Aβ levels in CSF samples of MS patients, albeit puzzling (Hein Née Maier et al., 2008; Valis et al., 2008; Sladkova et al., 2011; Szalardy et al., 2013), mostly evidenced that a general alteration of Aβ metabolism occurs in MS (Mattsson et al., 2009; Mai et al., 2011; Mori et al., 2011; Augutis et al., 2013).

In line with the possibility that inflammation-induced alteration of Aβ homeostasis could be a key factor in cognitive dysfunction, a study explored the correlation between CSF Aβ_1–42_ levels and TBS-induced plasticity in a group of cognitive impaired (CI) and cognitive preserved (CP) MS patients (Mori et al., 2011). It was found that Aβ_1–42_ levels were lower in CI patients compared to both CP patients and controls. Furthermore, CSF Aβ_1–42_ levels inversely correlated with the number of Gd+ lesions at MRI. Finally, altered iTBS-induced synaptic plasticity was observed in CI patients, and Aβ_1–42_ CSF levels positively correlated with reduced LTP-like plasticity. Overall, these data suggest that inflammation-driven alteration of Aβ metabolism in MS could disrupt LTP and impair cognitive function. Intriguingly, in AD experimental models specific proinflammatory cytokines could alter Aβ synthesis and clearance (Wang et al., 2015). In line with this, we propose that, as in AD, a possible imbalance between proinflammatory and anti-inflammatory cytokines leading to altered Aβ homeostasis may occur also in MS and contribute to the cognitive deficit observed in this disorder.

## Relation Between Proinflammatory Cytokines and Amyloid-β in MS Patients

To test the possibility that inflammation could interfere with Aβ metabolism in RR-MS patients, we investigated possible correlations between Aβ_1–42_ concentrations and the levels of some proinflammatory and anti-inflammatory molecules in the CSF (IL-1β, IL1-ra, IL-8, IL-10, IL12, TNFα, IFNγ). The study, involving 103 human subjects, was approved by the Ethics Committee of the University Hospital Tor Vergata, Rome. All patients gave written informed consent to take part to the study. The diagnosis of RR-MS was established according to published criteria (Polman et al., 2011). Clinical and demographic characteristics of MS patients are shown in Table 1. No immunoactive drug was given before hospitalization and corticosteroids or immune-modulating therapies were initiated later. Lumbar puncture was performed at the time of diagnosis, during hospitalization. CSF was centrifuged and immediately stored at −80C until analyzed using a Bio-Plex multiplex cytokine assay (Bio-Rad Laboratories, Hercules, CA, USA) according to the manufacturer’s instructions. For the analysis of Aβ_1–42_ standard procedures using commercially available sandwich enzyme-linked immunosorbent assays (Innotest β-Amyloid_1–42_, Innogenetics, Ghent, Belgium) were employed (Sancesario et al., 2010). For the analysis of cytokines levels, concentrations were calculated according to a standard curve generated for each target and expressed as pg/ml. An arbitrary value of 0 pg/ml was assigned to the concentrations of the cytokines measured below the detection threshold.

**Table 1:** Demographic and clinical characteristics of patients, correlations between Aβ_1–42_ and proinflammatory cytokines and anti-inflammatory molecules.

A			*N* = 103
Age	Mean (SD)		35 (10.3)
Sex, F	*n* (%)		69 (67%)
EDSS	Median (25–75th percentiles)		2 (1–2.5)
Disease duration	Median (25–75th percentiles)		12 (2–34)
**B**	**Aβ_1–42_**	***p***	**padj**.
IL-1β	−0.18	0.073	0.132
IL-8	−0.42	<0.001	<0.001
IL-10	0.38	<0.001	<0.001
IL-12	−0.01	0.954	0.954
IFNγ	−0.22	0.034	0.077
TNFα	−0.14	0.16	0.206
IL-1ra	0.34	0.001	0.002

*(A) Demographic and clinical characteristics of patients, (B) Spearman rho correlation coefficients between Aβ_1–42_ and cytokines. EDSS, Expanded Disability Status Scale; Aβ_1–42_, Amyloid-β_1–42_; IL, interleukin; IL-1ra, interleukin-1 receptor antagonist; TNFα, tumor necrosis factor α; IFNγ, interferon γ; p adj, p value after Benjamini–Hochberg correction*.

Data are presented as mean (standard deviation, SD) or as median (25–75th percentiles) if not normally distributed. Kolmogorov-Smirnov test was applied to verify normality of data distribution. Non parametric Spearman’s correlational analysis was performed to evaluate the correlation between CSF levels of Aβ_1–42_ and CSF levels of the main proinflammatory and anti-inflammatory cytokines. In addition, the correlation between CSF levels of Aβ_1–42_ and age, Expanded Disability Status Scale (EDSS) at baseline and disease duration was evaluated. Non parametric Mann-Whitney test was applied to evaluate difference between sexes in Aβ_1–42_ levels. A *p* value <0.05 was considered significant. Benjamini–Hochberg correction was applied to adjust the *p* value and control the false discovery rate in the multiple testing.

The results showed no significant correlations between CSF levels of Aβ_1–42_ and age (Spearman’r = −0.05, *p* = 0.780), disease duration (Spearman’r = −0.17, *p* = 0.149) and EDSS (Spearman’r = 0.02, *p* = 0.955). No significant differences between genders (pMann-Whitney = 0.519) and disease activity at diagnosis (pMann-Whitney = 0.520) were found. CSF levels of Aβ_1–42_ were negatively correlated with IL-8 (*r* = −0.417; *p* < 0.001) and IFNγ (*r* = −0.216; *p* = 0.034), borderline with IL1-β (*r* = −0.18; *p* = 0.073), and positively correlated with IL-10 (*r* = 0.381; *p* < 0.001) and IL-1ra (*r* = 0.341; *p* = 0.001; Table 1). Applying the Benjamini-Hochberg correction further confirmed a positive correlation between Aβ_1–42_ and IL-8 (padj < 0.001), IL10 (padj < 0.001) and IL1-ra (padj = 0.002), whereas correlation with IFNγ was borderline (*p* = 0.077) and correlation with IL-1β was no longer significant (*p* = 0.132). Correlations between Aβ_1–42_ levels and TNFα and IL-12 were not significant.

## Conclusion

MS is classically considered a demyelinating disease of the CNS, primarily involving the white matter and followed by neurodegeneration in the late phases. Cognitive deficits in MS have been related to white matter damage, albeit hippocampal structures involvement is emerging as a key component of the cognitive dysfunction observed both in EAE and MS. In recent years, neuroinflammation has been identified as a factor inducing both neurodegeneration and synaptic plasticity dysfunction. How inflammation alters hippocampal plasticity in MS is still scarcely understood, although it has been proposed that altered Aβ metabolism could play a crucial role.

Our data add novelty to previous evidence showing that inflammation influences Aβ metabolism in MS. The significant negative correlation between CSF Aβ_1–42_ concentrations and the proinflammatory cytokines IL-8 and IFNγ suggests that inflammatory response is associated to dysregulation of Aβ synthesis and degradation. IL-8 is a well-known biomarker of neuroinflammation (Komori et al., 2015) and its CSF levels have been found elevated in several inflammatory and non-inflammatory neurological conditions (Bielekova et al., 2012). In MS, a previous study investigated the association between IL-8 CSF levels and visual recovery 6 months after acute optic neuritis, showing that higher levels of IL-8 correlated with incomplete visual recovery (Rossi et al., 2014). In a further study, elevated IL-8 CSF levels during acute inflammation correlated with clinical progression in patients with radiologically isolated syndrome and with the risk to develop MS in those with clinically isolated syndrome (Rossi et al., 2015). IFNγ has been involved in the pathophysiology of both MS and EAE. Indeed, higher IFNγ levels within the CNS have been found during inflammation in MS (Cannella and Raine, 1995; Kahl et al., 2002) and also in EAE (Gardner et al., 2013; Hidaka et al., 2014).

Our results also show that anti-inflammatory cytokines may have a beneficial effect contributing to reduce the alterations of Aβ metabolism. In particular, IL-10 is considered one of the main anti-inflammatory cytokines involved in modulating brain inflammatory response (Kwilasz et al., 2015) and has been proposed as a useful treatment in several neurological conditions characterized by persistent neuroinflammation and neurodegeneration (Cua et al., 2001; Joniec-Maciejak et al., 2014). IL-1ra, is an anti-inflammatory endogenous molecule acting as competitive inhibitor of IL-1β (Seckinger et al., 1987). Notably, IL-1ra administration ameliorated EAE clinical manifestations (Martin and Near, 1995; Badovinac et al., 1998; Furlan et al., 2007). These results suggest that anti-inflammatory molecules may reduce the impact of neuroinflammation on Aβ_1–42_ homeostasis, in line with previous results showing that altered hippocampal synaptic plasticity in EAE could be reduced by blocking IL-1β transmission (Mori et al., 2014).

**Figure 1:**
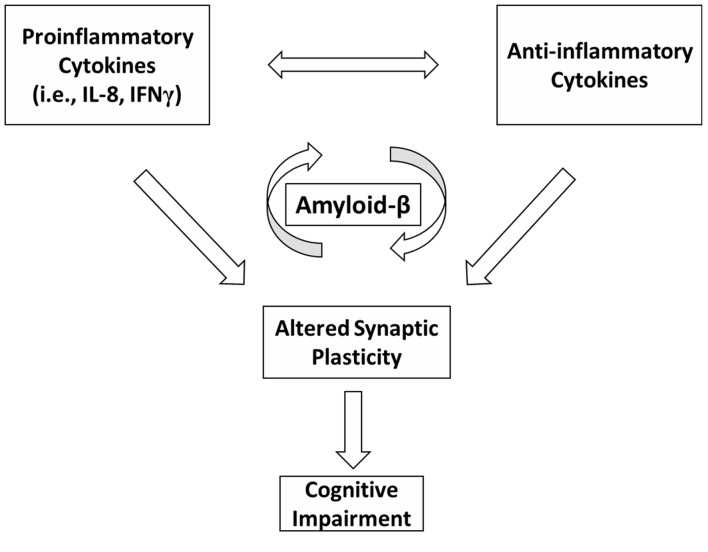
A schematic model depicting possible interactions between inflammation, Amyloid-β (Aβ) metabolism, synaptic plasticity and cognitive dysfunction in multiple sclerosis (MS). In MS, an imbalance between proinflammatory and anti-inflammatory cytokines may differently affect Aβ metabolism. Inflammation is also able to alter synaptic plasticity. Altered plasticity could be a possible substrate for cognitive deficits. Altered Aβ homeostasis may represent a key factor linking together inflammation, synaptic plasticity and cognitive impairment in MS.

Our data suggest that altered Aβ homeostasis could represent a key factor linking together inflammation, synaptic plasticity and cognitive dysfunction in MS (Figure 1). Although previous reports have shown an association between IL-1β and Aβ homeostasis in AD experimental models (Wang et al., 2015), this correlation was not found in the MS patients involved in the present study. However, on the basis of the preliminary data reported here, we may conclude that the inflammatory process underlying MS is complex and may implicate a more widespread cytokine release. Additional investigations are required to explore the relationship between CSF chemokines and Aβ metabolism in normal subjects and to further characterize the role of CSF inflammation and altered synaptic plasticity in the development of cognitive deficits in MS patients.

In conclusion, the differential modulation of Aβ metabolism by proinflammatory and anti-inflammatory cytokines could be relevant to identify novel therapeutic approaches to hinder the progression of the cognitive dysfunction in MS.

## Author Contributions

MSB: data interpretation, work conception and design, drafting the work, work revision; SG and LG: data interpretation, work revision; GAM: design of the work, work revision; IS: data analysis, data interpretation, work revision; AF and RF: data analysis, data interpretation; GMS: data acquisition and analysis, data interpretation; JDG and MS: data acquisition and analysis; FM: data acquisition and analysis, data interpretation; DC: data interpretation, work conception and design, work revision; EI: work conception and design, drafting the work, work revision.

## Conflict of Interest Statement

The authors declare that the research was conducted in the absence of any commercial or financial relationships that could be construed as a potential conflict of interest.

## References

[B1] Andreasson U. Portelius E. Andersson M. E. Blennow K. Zetterberg H. (2007). Aspects of β-amyloid as a biomarker for Alzheimer’s disease. Biomark. Med. 1, 59–78. 10.2217/17520363.1.1.59 20477461

[B2] Augutis K. Axelsson M. Portelius E. Brinkmalm G. Andreasson U. Gustavsson M. K.. (2013). Cerebrospinal fluid biomarkers of β-amyloid metabolism in multiple sclerosis. Mult. Scler. 19, 543–552. 10.1177/1352458512460603 23069872

[B3] Badovinac V. Mostarica-Stojkovićc M. Dinarello C. A. Stošic-Grujičic S. (1998). Interleukin-1 receptor antagonist suppresses experimental autoimmune encephalomyelitis (EAE) in rats by influencing the activation and proliferation of encephalitogenic cells. J. Neuroimmunol. 85, 87–95. 10.1016/s0165-5728(98)00020-4 9627001

[B4] Benedict R. H. B. Cookfair D. Gavett R. Gunther M. Munschauer F. Garg N.. (2006). Validity of the minimal assessment of cognitive function in multiple sclerosis (MACFIMS). J. Int. Neuropsychol. Soc. 12, 549–558. 10.1017/s1355617706060723 16981607

[B5] Bielekova B. Komori M. Xu Q. Reich D. S. Wu T. (2012). Cerebrospinal fluid IL-12p40, CXCL13 and IL-8 as a combinatorial biomarker of active intrathecal inflammation. PLoS One 7:e48370. 10.1371/journal.pone.0048370 23226202 PMC3511462

[B6] Blennow K. Hampel H. (2003). CSF markers for incipient Alzheimer’s disease. Lancet Neurol. 2, 605–613. 10.1016/s1474-4422(03)00530-1 14505582

[B7] Bliss T. V. P. Collingridge G. L. (1993). A synaptic model of memory: long-term potentiation in the hippocampus. Nature 361, 31–39. 10.1038/361031a0 8421494

[B8] Calabrese M. Agosta F. Rinaldi F. Mattisi I. Grossi P. Favaretto A.. (2009). Cortical lesions and atrophy associated with cognitive impairment in relapsing-remitting multiple sclerosis. Arch. Neurol. 66, 1144–1150. 10.1001/archneurol.2009.174 19752305

[B9] Cannella B. Raine C. S. (1995). The adhesion molecule and cytokine profile of multiple sclerosis lesions. Ann. Neurol. 37, 424–435. 10.1002/ana.410370404 7536402

[B10] Caramia M. D. Palmieri M. G. Desiato M. T. Boffa L. Galizia P. Rossini P. M.. (2004). Brain excitability changes in the relapsing and remitting phases of multiple sclerosis: a study with transcranial magnetic stimulation. Clin. Neurophysiol. 115, 956–965. 10.1016/j.clinph.2003.11.024 15003779

[B11] Carmona O. Masuet C. Santiago O. Alía P. Moral E. Alonso-Magdalena L.. (2011). Multiple sclerosis and cognitive decline: is ApoE-4 a surrogate marker? Acta Neurol. Scand. 124, 258–263. 10.1111/j.1600-0404.2010.01473.x 21208197

[B12] Centonze D. Muzio L. Rossi S. Cavasinni F. De Chiara V. Bergami A.. (2009). Inflammation triggers synaptic alteration and degeneration in experimental autoimmune encephalomyelitis. J. Neurosci. 29, 3442–3452. 10.1523/jneurosci.5804-08.2009 19295150 PMC6665268

[B13] Chen Q. S. Wei W. Z. Shimahara T. Xie C. W. (2002). Alzheimer amyloid β-peptide inhibits the late phase of long-term potentiation through calcineurin-dependent mechanisms in the hippocampal dentate gyrus. Neurobiol. Learn. Mem. 77, 354–371. 10.1006/nlme.2001.4034 11991763

[B14] Chiaravalloti N. D. DeLuca J. (2008). Cognitive impairment in multiple sclerosis. Lancet Neurol. 7, 1139–1151. 10.1016/S1474-4422(08)70259-X 19007738

[B15] Coebergh J. Roosendaal S. Polman C. Geurts J. van Woerkom T. (2010). Acute severe memory impairment as a presenting symptom of multiple sclerosis: a clinical case study with 3D double inversion recovery MR imaging. Mult. Scler. 16, 1521–1524. 10.1177/1352458510383302 20846999

[B16] Cua D. J. Hutchins B. La Face D. M. Stohlman S. A. Coffman R. L. (2001). Central nervous system expression of IL-10 inhibits autoimmune encephalomyelitis. J. Immunol. 166, 602–608. 10.4049/jimmunol.166.1.602 11123343

[B17] Cunningham A. J. Murray C. A. O’Neill L. A. Lynch M. A. O’Connor J. J. (1996). Interleukin-1β (IL-1β) and tumour necrosis factor (TNF) inhibit long-term potentiation in the rat dentate gyrus *in vitro*. Neurosci. Lett. 203, 17–20. 10.1016/0304-3940(95)12252-4 8742036

[B18] D’Intino G. Paradisi M. Fernandez M. Giuliani A. Aloe L. Giardino L.. (2005). Cognitive deficit associated with cholinergic and nerve growth factor down-regulation in experimental allergic encephalomyelitis in rats. Proc. Natl. Acad. Sci. U S A 102, 3070–3075. 10.1073/pnas.0500073102 15710875 PMC548798

[B19] Dal Canto M. C. Lipton H. L. (1975). Primary demyelination in Theiler’s virus infection. An ultrastructural study. Lab. Invest. 33, 626–637. 1202282

[B20] Deloire M. S. Bonnet M. C. Salort E. Arimone Y. Boudineau M. Petry K. G.. (2006). How to detect cognitive dysfunction at early stages of multiple sclerosis? Mult. Scler. 12, 445–452. 10.1191/1352458506ms1289oa 16900758

[B21] Di Filippo M. Chiasserini D. Gardoni F. Viviani B. Tozzi A. Giampà C.. (2013). Effects of central and peripheral inflammation on hippocampal synaptic plasticity. Neurobiol. Dis. 52, 229–236. 10.1016/j.nbd.2012.12.009 23295855

[B22] Di Filippo M. de Iure A. Giampà C. Chiasserini D. Tozzi A. Orvietani P. L.. (2016). Persistent activation of microglia and NADPH oxidase drive hippocampal dysfunction in experimental multiple sclerosis. Sci. Rep. 6:20926. 10.1038/srep20926 26887636 PMC4757867

[B23] Di Lazzaro V. Pilato F. Saturno E. Oliviero A. Dileone M. Mazzone P.. (2005). Theta-burst repetitive transcranial magnetic stimulation suppresses specific excitatory circuits in the human motor cortex. J. Physiol. 565, 945–950. 10.1113/jphysiol.2005.087288 15845575 PMC1464561

[B24] Ferguson B. Matyszak M. K. Esiri M. M. Perry V. H. (1997). Axonal damage in acute multiple sclerosis lesions. Brain 120, 393–399. 10.1093/brain/120.3.393 9126051

[B25] Fitzgerald P. B. Brown T. L. Marston N. A. U. Oxley T. De Castella A. Daskalakis Z. J.. (2004). Reduced plastic brain responses in schizophrenia: a transcranial magnetic stimulation study. Schizophr. Res. 71, 17–26. 10.1016/j.schres.2004.01.018 15374568

[B26] Furlan R. Bergami A. Brambilla E. Butti E. De Simoni M. G. Campagnoli M.. (2007). HSV-1-mediated IL-1 receptor antagonist gene therapy ameliorates MOG(35–55)-induced experimental autoimmune encephalomyelitis in C57BL/6 mice. Gene Ther. 14, 93–98. 10.1038/sj.gt.3302805 16929354

[B27] Gardner C. Magliozzi R. Durrenberger P. F. Howell O. W. Rundle J. Reynolds R. (2013). Cortical grey matter demyelination can be induced by elevated pro-inflammatory cytokines in the subarachnoid space of MOG-immunized rats. Brain 136, 3596–3608. 10.1093/brain/awt279 24176976

[B28] Garg S. K. Kipnis J. Banerjee R. (2009). IFN-γ and IL-4 differentially shape metabolic responses and neuroprotective phenotype of astrocytes. J. Neurochem. 108, 1155–1166. 10.1111/j.1471-4159.2009.05872.x 19141080

[B29] Gentile A. Mori F. Bernardini S. Centonze D. (2015). Role of amyloid-β CSF levels in cognitive deficit in MS. Clin. Chim. Acta 449, 23–30. 10.1016/j.cca.2015.01.035 25659291

[B30] Griffin W. S. T. Liu L. Li Y. Mrak R. E. Barger S. W. (2006). Interleukin-1 mediates Alzheimer and Lewy body pathologies. J. Neuroinflammation 3:5. 10.1186/1742-2094-3-5 16542445 PMC1435743

[B31] Hein Née Maier K. Köhler A. Diem R. Sättler M. B. Demmer I. Lange P.. (2008). Biological markers for axonal degeneration in CSF and blood of patients with the first event indicative for multiple sclerosis. Neurosci. Lett. 436, 72–76. 10.1016/j.neulet.2008.02.064 18359164

[B32] Hickman S. E. Allison E. K. El Khoury J. (2008). Microglial dysfunction and defective β-amyloid clearance pathways in aging Alzheimer’s disease mice. J. Neurosci. 28, 8354–8360. 10.1523/jneurosci.0616-08.2008 18701698 PMC2597474

[B33] Hidaka Y. Inaba Y. Matsuda K. Itoh M. Kaneyama T. Nakazawa Y.. (2014). Cytokine production profiles in chronic relapsing-remitting experimental autoimmune encephalomyelitis: IFN-γ and TNF-α are important participants in the first attack but not in the relapse. J. Neurol. Sci. 340, 117–122. 10.1016/j.jns.2014.02.039 24655735

[B34] Huang Y. Edwards M. J. Rounis E. Bhatia K. P. Rothwell J. C. (2005). Theta burst stimulation of the human motor cortex. Neuron 45, 201–206. 10.1016/j.neuron.2004.12.033 15664172

[B35] Ivanov S. Lindén A. (2009). Interleukin-17 as a drug target in human disease. Trends Pharmacol. Sci. 30, 95–103. 10.1016/j.tips.2008.11.004 19162337

[B36] Iwata N. Tsubuki S. Takaki Y. Shirotani K. Lu B. Gerard N. P.. (2001). Metabolic regulation of brain Abeta by neprilysin. Science 292, 1550–1552. 10.1126/science.1059946 11375493

[B37] Joniec-Maciejak I. Ciesielska A. Wawer A. Sznejder-Pachołek A. Schwenkgrub J. Cudna A.. (2014). The influence of AAV2-mediated gene transfer of human IL-10 on neurodegeneration and immune response in a murine model of Parkinson’s disease. Pharmacol. Rep. 66, 660–669. 10.1016/j.pharep.2014.03.008 24948069

[B38] Kahl K. G. Kruse N. Faller H. Weiss H. Rieckmann P. (2002). Expression of tumor necrosis factor-α and interferon-γ mRNA in blood cells correlates with depression scores during an acute attack in patients with multiple sclerosis. Psychoneuroendocrinology 27, 671–681. 10.1016/s0306-4530(01)00068-3 12084660

[B39] Kessels H. W. Malinow R. (2009). Synaptic AMPA receptor plasticity and behavior. Neuron 61, 340–350. 10.1016/j.neuron.2009.01.015 19217372 PMC3917551

[B40] Kim J. Basak J. M. Holtzman D. M. (2009). The role of apolipoprotein E in Alzheimer’s disease. Neuron 63, 287–303. 10.1016/j.neuron.2009.06.026 19679070 PMC3044446

[B41] Kincses Z. T. Ropele S. Jenkinson M. Khalil M. Petrovic K. Loitfelder M.. (2011). Lesion probability mapping to explain clinical deficits and cognitive performance in multiple sclerosis. Mult. Scler. 17, 681–689. 10.1177/1352458510391342 21177325

[B42] Klyubin I. Walsh D. M. Lemere C. A. Cullen W. K. Shankar G. M. Betts V.. (2005). Amyloid beta protein immunotherapy neutralizes Abeta oligomers that disrupt synaptic plasticity *in vivo*. Nat. Med. 11, 556–561. 10.1038/nm1234 15834427

[B43] Komori M. Blake A. Greenwood M. Lin Y. C. Kosa P. Ghazali D.. (2015). Cerebrospinal fluid markers reveal intrathecal inflammation in progressive multiple sclerosis. Ann. Neurol. 78, 3–20. 10.1002/ana.24408 25808056 PMC5568079

[B44] Krasemann S. Madore C. Cialic R. Baufeld C. Calcagno N. El Fatimy R.. (2017). The TREM2-APOE pathway drives the transcriptional phenotype of dysfunctional microglia in neurodegenerative diseases. Immunity 47, 566.e9–581.e9. 10.1016/j.immuni.2017.08.008 28930663 PMC5719893

[B45] Kwilasz A. J. Grace P. M. Serbedzija P. Maier S. F. Watkins L. R. (2015). The therapeutic potential of interleukin-10 in neuroimmune diseases. Neuropharmacology 96, 55–69. 10.1016/j.neuropharm.2014.10.020 25446571 PMC5144739

[B46] Lassmann H. (2011). Mechanisms of neurodegeneration shared between multiple sclerosis and Alzheimer’s disease. J. Neural Transm. 118, 747–752. 10.1007/s00702-011-0607-8 21373761

[B47] Li S. Hong S. Shepardson N. E. Walsh D. M. Shankar G. M. Selkoe D. (2009). Soluble oligomers of amyloid β protein facilitate hippocampal long-term depression by disrupting neuronal glutamate uptake. Neuron 62, 788–801. 10.1016/j.neuron.2009.05.012 19555648 PMC2702854

[B48] Linker R. A. Sendtner M. Gold R. (2005). Mechanisms of axonal degeneration in EAE—lessons from CNTF and MHC I knockout mice. J. Neurol. Sci. 233, 167–172. 10.1016/j.jns.2005.03.021 15949503

[B49] Mai W. Hu X. Lu Z. Peng F. Wang Y. (2011). Cerebrospinal fluid levels of soluble amyloid precursor protein and β-amyloid 42 in patients with multiple sclerosis, neuromyelitisoptica and clinically isolated syndrome. J. Int. Med. Res. 39, 2402–2413. 10.1177/147323001103900641 22289560

[B50] Malenka R. C. (2003). Opinion: the long-term potential of LTP. Nat. Rev. Neurosci. 4, 923–926. 10.1038/nrn1258 14595403

[B51] Mandolesi G. Musella A. Gentile A. Grasselli G. Haji N. Sepman H. (2013). Interleukin-1 alters glutamate transmission at Purkinje cell synapses in a mouse model of multiple sclerosis. J. Neurosci. 33, 12105–12121. 10.1523/jneurosci.5369-12.2013 23864696 PMC6794065

[B52] Mariorenzi R. Zarola F. Caramia M. D. Paradiso C. Rossini P. M. (1991). Non-invasive evaluation of central motor tract excitability changes following peripheral nerve stimulation in healthy humans. Electroencephalogr. Clin. Neurophysiol. 81, 90–101. 10.1016/0168-5597(91)90002-f 1708719

[B53] Martin D. Near S. L. (1995). Protective effect of the interleukin-1 receptor antagonist (IL-1ra) on experimental allergic encephalomyelitis in rats. J. Neuroimmunol. 61, 241–245. 10.1016/0165-5728(95)00108-e 7593560

[B54] Masters C. L. Selkoe D. J. (2012). Biochemistry of amyloid β-protein and amyloid deposits in Alzheimer disease. Cold Spring Harb. Perspect. Med. 2:a006262. 10.1101/cshperspect.a006262 22675658 PMC3367542

[B55] Mattsson N. Axelsson M. Haghighi S. Malmeström C. Wu G. Anckarsäter R.. (2009). Reduced cerebrospinal fluid BACE1 activity in multiple sclerosis. Mult. Scler. 15, 448–454. 10.1177/1352458508100031 19153172

[B56] Miller S. D. (1995). Pathogenesis of Theiler’s murine encephalomyelitis virus-induced demyelinating disease-a model of multiple sclerosis. ACLAD Newslett. 16, 4–6.

[B57] Minichiello L. (2009). TrkBsignalling pathways in LTP and learning. Nat. Rev. Neurosci. 10, 850–860. 10.1038/nrn2738 19927149

[B58] Morgese M. G. Colaianna M. Mhillaj E. Zotti M. Schiavone S. D’Antonio P.. (2015). Soluble beta amyloid evokes alteration in brain norepinephrine levels: role of nitric oxide and interleukin-1. Front. Neurosci. 9:428. 10.3389/fnins.2015.00428 26594145 PMC4633524

[B59] Mori F. Kusayanagi H. Buttari F. Centini B. Monteleone F. Nicoletti C. G.. (2012). Early treatment with high-dose interferon beta-1a reverses cognitive and cortical plasticity deficits in multiple sclerosis. Funct. Neurol. 27, 163–168. 23402677 PMC3812766

[B60] Mori F. Nisticò R. Mandolesi G. Piccinin S. Mango D. Kusayanagi H.. (2014). Interleukin-1β promotes long-term potentiation in patients with multiple sclerosis. NeuroMolecular Med. 16, 38–51. 10.1007/s12017-013-8249-7 23892937

[B61] Mori F. Nisticò R. Nicoletti C. G. Zagaglia S. Mandolesi G. Piccinin S.. (2016). RANTES correlates with inflammatory activity and synaptic excitability in multiple sclerosis. Mult. Scler. 22, 1405–1412. 10.1177/1352458515621796 26733422

[B62] Mori F. Rossi S. Sancesario G. Codecà C. Mataluni G. Monteleone F.. (2011). Cognitive and cortical plasticity deficits correlate with altered amyloid-β CSF levels in multiple sclerosis. Neuropsychopharmacology 36, 559–568. 10.1038/npp.2010.187 20944553 PMC3055691

[B63] Murray C. A. Lynch M. A. (1998). Evidence that increased hippocampal expression of the cytokine interleukin-1 beta is a common trigger for age- and stress-induced impairments in long-term potentiation. J. Neurosci. 18, 2974–2981. 9526014 10.1523/JNEUROSCI.18-08-02974.1998 PMC6792583

[B64] Nisticò R. Mango D. Mandolesi G. Piccinin S. Berretta N. Pignatelli M.. (2013). Inflammation subverts hippocampal synaptic plasticity in experimental multiple sclerosis. PLoS One 8:e54666. 10.1371/journal.pone.0054666 23355887 PMC3552964

[B65] Olivares T. Nieto A. Sánchez M. P. Wollmann T. Hernández M. A. Barroso J. (2005). Pattern of neuropsychological impairment in the early phase of relapsing-remitting multiple sclerosis. Mult. Scler. 11, 191–197. 10.1191/1352458505ms1139oa 15794394

[B66] Pardini M. Uccelli A. Grafman J. Yaldizli Ö. Mancardi G. Roccatagliata L. (2014). Isolated cognitive relapses in multiple sclerosis. J. Neurol. Neurosurg. Psychiatry 85, 1035–1037. 10.1136/jnnp-2013-307275 24686566

[B300] Polman C. H. Reingold S. C. Banwell B. Clanet M. Cohen J. A. Filippi M.. (2011). Diagnostic criteria for multiple sclerosis: 2010 revisions to the McDonald criteria. Ann. Neurol. 69, 292–302. 10.1002/ana.22366 21387374 PMC3084507

[B67] Portaccio E. Goretti B. Zipoli V. Nacmias B. Stromillo M. L. Bartolozzi M. L.. (2009). APOE-epsilon4 is not associated with cognitive impairment in relapsing-remitting multiple sclerosis. Mult. Scler. 15, 1489–1494. 10.1177/1352458509348512 19965518

[B68] Rao S. M. Leo G. J. Ellington L. Nauertz T. Bernardin L. Unverzagt F. (1991). Cognitive dysfunction in multiple sclerosis. II. Impact on employment and social functioning. Neurology 41, 692–696. 10.1212/WNL.41.5.692 1823781

[B70] Rossi S. Furlan R. De Chiara V. Motta C. Studer V. Mori F.. (2012a). Interleukin-1β causes synaptic hyperexcitability in multiple sclerosis. Ann. Neurol. 71, 76–83. 10.1002/ana.22512 22275254

[B74] Rossi S. Studer V. Motta C. De Chiara V. Barbieri F. Bernardi G.. (2012b). Inflammation inhibits GABA transmission in multiple sclerosis. Mult. Scler. 18, 1633–1635. 10.1177/1352458512440207 22419673

[B69] Rossi F. Giorgio A. Battaglini M. Stromillo M. L. Portaccio E. Goretti B.. (2012). Relevance of brain lesion location to cognition in relapsing multiple sclerosis. PLoS One 7:e44826. 10.1371/journal.pone.0044826 23144775 PMC3489883

[B71] Rossi S. Mancino R. Bergami A. Mori F. Castelli M. De Chiara V.. (2011). Potential role of IL-13 in neuroprotection and cortical excitability regulation in multiple sclerosis. Mult. Scler. 17, 1301–1312. 10.1177/1352458511410342 21677024

[B72] Rossi S. Motta C. Studer V. Macchiarulo G. Germani G. Finardi A.. (2015). Subclinical central inflammation is risk for RIS and CIS conversion to MS. Mult. Scler. 21, 1443–1452. 10.1177/1352458514564482 25583841

[B73] Rossi S. Motta C. Studer V. Rocchi C. Macchiarulo G. Barbieri F.. (2014). Interleukin-8 is associated with acute and persistent dysfunction after optic neuritis. Mult. Scler. 20, 1841–1850. 10.1177/1352458514537365 24876157

[B75] Saito T. Iwata N. Tsubuki S. Takaki Y. Takano J. Huang S.-M.. (2005). Somatostatin regulates brain amyloid beta peptide Abeta42 through modulation of proteolytic degradation. Nat. Med. 11, 434–439. 10.1038/nm1206 15778722

[B76] Sancesario G. M. Esposito Z. Nuccetelli M. Bernardini S. Sorge R. Martorana A.. (2010). Aβ1–42 detection in CSF of Alzheimer’s disease is influenced by temperature: indication of reversible Aβ1–42 aggregation? Exp. Neurol. 223, 371–376. 10.1016/j.expneurol.2009.07.028 19664624

[B77] Schmidt J. Barthel K. Wrede A. Salajegheh M. Bähr M. Dalakas M. C. (2008). Interrelation of inflammation and APP in sIBM: IL-1β induces accumulation of β-amyloid in skeletal muscle. Brain 131, 1228–1240. 10.1093/brain/awn053 18420712 PMC2367696

[B78] Seckinger P. Lowenthal J. W. Williamson K. Dayer J. M. MacDonald H. R. (1987). A urine inhibitor of interleukin 1 activity that blocks ligand binding. J. Immunol. 139, 1546–1549. 2957429

[B79] Shankar G. M. Li S. Mehta T. H. Garcia-Munoz A. Shepardson N. E. Smith I.. (2008). Amyloid-β protein dimers isolated directly from Alzheimer’s brains impair synaptic plasticity and memory. Nat. Med. 14, 837–842. 10.1038/nm1782 18568035 PMC2772133

[B80] Shi J. Zhao C. B. Vollmer T. L. Tyry T. M. Kuniyoshi S. M. (2008). APOE epsilon 4 allele is associated with cognitive impairment in patients with multiple sclerosis. Neurology 70, 185–190. 10.1212/01.WNL.0000264004.62612.44 17460153

[B81] Sicotte N. L. Kern K. C. Giesser B. S. Arshanapalli A. Schultz A. Montag M.. (2008). Regional hippocampal atrophy in multiple sclerosis. Brain 131, 1134–1141. 10.1093/brain/awn030 18375977

[B82] Sladkova V. Mareš J. Lubenova B. Zapletalova J. Stejskal D. Hlustik P.. (2011). Degenerative and inflammatory markers in the cerebrospinal fluid of multiple sclerosis patients with relapsing-remitting course of disease and after clinical isolated syndrome. Neurol. Res. 33, 415–420. 10.1179/016164110X12816242542535 21535941

[B83] S-Rózsa K. Rubakhin S. S. Szücs A. Hughes T. K. Stefano G. B. (1997). Opposite effects of interleukin-2 and interleukin-4 on GABA-induced inward currents of dialysed Lymnaea neurons. Gen. Pharmacol. 29, 73–77. 10.1016/s0306-3623(96)00527-7 9195196

[B84] Stampanoni Bassi M. Mori F. Buttari F. Marfia G. A. Sancesario A. Centonze D.. (2017). Neurophysiology of synaptic functioning in multiple sclerosis. Clin. Neurophysiol. 128, 1148–1157. 10.1016/j.clinph.2017.04.006 28511127

[B85] Stuchlik A. (2014). Dynamic learning and memory, synaptic plasticity and neurogenesis: an update. Front. Behav. Neurosci. 8:106. 10.3389/fnbeh.2014.00106 24744707 PMC3978286

[B86] Szalardy L. Zadori D. Simu M. Bencsik K. Vecsei L. Klivenyi P. (2013). Evaluating biomarkers of neuronal degeneration and neuroinflammation in CSF of patients with multiple sclerosis-osteopontin as a potential marker of clinical severity. J. Neurol. Sci. 331, 38–42. 10.1016/j.jns.2013.04.024 23706476

[B87] Trapp B. D. Peterson J. Ransohoff R. M. Rudick R. Mörk S. Bö L. (1998). Axonal transection in the lesions of multiple sclerosis. N. Engl. J. Med. 338, 278–285. 10.1056/nejm199801293380502 9445407

[B88] Tu J. L. Zhao C. B. Vollmer T. Coons S. Lin H. J. Marsh S.. (2009). APOE 4 polymorphism results in early cognitive deficits in an EAE model. Biochem. Biophys. Res. Commun. 384, 466–470. 10.1016/j.bbrc.2009.04.153 19422789

[B89] Valis M. Talab R. Stourac P. Andrys C. Masopust J. (2008). Tau protein, phosphorylated tau protein and beta-amyloid42 in the cerebrospinal fluid of multiple sclerosis patients. Neuro Endocrinol. Lett. 29, 971–976. 19112391

[B90] Vellinga M. M. Geurts J. J. G. Rostrup E. Uitdehaag B. M. J. Polman C. H. Barkhof F.. (2009). Clinical correlations of brain lesion distribution in multiple sclerosis. J. Magn. Reson. Imaging 29, 768–773. 10.1002/jmri.21679 19306365

[B91] Walsh D. M. Klyubin I. Fadeeva J. V. Cullen W. K. Anwyl R. Wolfe M. S.. (2002). Naturally secreted oligomers of amyloid β protein potently inhibit hippocampal long-term potentiation *in vivo*. Nature 416, 535–539. 10.1038/416535a 11932745

[B92] Wang W.-Y. Tan M.-S. Yu J.-T. Tan L. (2015). Role of proinflammatory cytokines released from microglia in Alzheimer’s disease. Ann. Transl. Med. 3:136. 10.3978/j.issn.2305-5839.2015.03.49 26207229 PMC4486922

[B93] Yamin G. (2009). NMDA receptor-dependent signaling pathways that underlie amyloid β-protein disruption of LTP in the hippocampus. J. Neurosci. Res. 87, 1729–1736. 10.1002/jnr.21998 19170166

[B94] Yirmiya R. Goshen I. (2011). Immune modulation of learning, memory, neural, plasticity and neurogenesis. Brain Behav. Immun. 25, 181–213. 10.1016/j.bbi.2010.10.015 20970492

[B95] Zeis T. Graumann U. Reynolds R. Schaeren-Wiemers N. (2008). Normal-appearing white matter in multiple sclerosis is in a subtle balance between inflammation and neuroprotection. Brain 131, 288–303. 10.1093/brain/awm291 18056737

[B96] Zhao D. Watson J. B. Xie C.-W. (2004). Amyloid beta prevents activation of calcium/calmodulin-dependent protein kinase II and AMPA receptor phosphorylation during hippocampal long-term potentiation. J. Neurophysiol. 92, 2853–2858. 10.1152/jn.00485.2004 15212428

[B97] Zhou Z. Peng X. Insolera R. Fink D. J. Mata M. (2009). IL-10 promotes neuronal survival following spinal cord injury. Exp. Neurol. 220, 183–190. 10.1016/j.expneurol.2009.08.018 19716366 PMC2788918

[B98] Ziehn M. O. Avedisian A. A. Tiwari-Woodruff S. Voskuhl R. R. (2010). Hippocampal CA1 atrophy and synaptic loss during experimental autoimmune encephalomyelitis, EAE. Lab. Invest. 90, 774–786. 10.1038/labinvest.2010.6 20157291 PMC3033772

[B99] Ziemann U. Paulus W. Nitsche M. A. Pascual-Leone A. Byblow W. D. Berardelli A.. (2008). Consensus: motor cortexplasticityprotocols. Brain Stimul. 1, 164–182. 10.1016/j.brs.2008.06.006 20633383

[B100] Zivadinov R. Sepcic J. Nasuelli D. De Masi R. Bragadin L. M. Tommasi M. A.. (2001). A longitudinal study of brain atrophy and cognitive disturbances in the early phase of relapsing-remitting multiple sclerosis. J. Neurol. Neurosurg. Psychiatry 70, 773–780. 10.1136/jnnp.70.6.773 11385012 PMC1737379

